# Monoolein Assisted Oil-Based Transdermal Delivery of Powder Vaccine

**DOI:** 10.3390/pharmaceutics12090814

**Published:** 2020-08-27

**Authors:** Momoko Kitaoka, Atsushi Oka, Masahiro Goto

**Affiliations:** 1 Department of Applied Chemistry, Graduate School of Engineering, Kyushu University, Fukuoka 819-0395, Japan; mkitaoka@mail.cstm.kyushu-u.ac.jp (M.K.); atsushioka.315918@gmail.com (A.O.); 2 Advanced Transdermal Drug Delivery System Center, Kyushu University, Fukuoka 819-0395, Japan; 3 Center for Future Chemistry, Kyushu University, Fukuoka 819-0395, Japan

**Keywords:** transdermal vaccine delivery, skin penetration enhancer, monoolein, non-invasive administration, oil-based formulation

## Abstract

An increasing number of protein vaccines have been researched for cancer, inflammation, and allergy therapies. Most of the protein therapeutics are administered through injection because orally-administered proteins are metabolized by the digestive system. Although transdermal administration has received increasing attention, the natural barrier formed by the skin is an obstacle. Monoolein is a common skin penetration enhancer that facilitates topical and transdermal drug delivery. Conventionally, it has been used in an aqueous vehicle, often with polyhydric alcohols. In the current study, monoolein was dissolved in an oil vehicle, isopropyl myristate, to facilitate the skin permeation of powder proteins. The skin permeabilities of the proteins were examined in-vivo and ex-vivo. Monoolein concentration-dependently enhanced the skin permeation of proteins. The protein permeability correlated with the zeta potential of the macromolecules. Dehydration of the stratum corneum (SC), lipid extraction from the SC, and disordering of ceramides caused by monoolein were demonstrated through Fourier transform infrared spectroscopic analysis and small-angle X-ray scattering analysis. An antigen model protein, ovalbumin from egg white, was delivered to immune cells in living mice, and induced antigen-specific IgG antibodies. The patch system showed the potential for transdermal vaccine delivery.

## 1. Introduction

Proteins as therapeutics include hormones, antibodies, and antigens that are necessary for the treatment of genetic disorders, autoimmunity/inflammation, cancers, and allergic diseases [1,2]. Protein drugs are expected to treat symptoms that cannot be effectively treated using existing small-molecule drugs, and increasing numbers are being developed and approved. Antigen proteins have larger molecular weights compared to that of conventional chemical therapeutics. Moreover, protein drugs require the preservation of the conformation and tertiary structures to retain the therapeutic activity. Protein drugs, including vaccines, have been conventionally administered through injection to avoid gastrointestinal digestion. However, the invasive approach reduces patient compliance, and needle-stick related issues are of concern. To address these issues, transdermal protein delivery is gaining an increasing attention because the transdermal route enables slow and sustained administration [3]. Self-administration of protein delivery patches is also possible, and the patch system is applicable to patients with gastrointestinal disorders. Moreover, prompt discontinuation of a drug is possible in response to undesired adverse events. However, the stratum corneum (SC) in the skin is an obstacle. The SC is composed of lipids and corneocytes and interrupts the invasion of hydrophilic macromolecules, viruses, and germs under normal circumstances.

**Figure 1:**
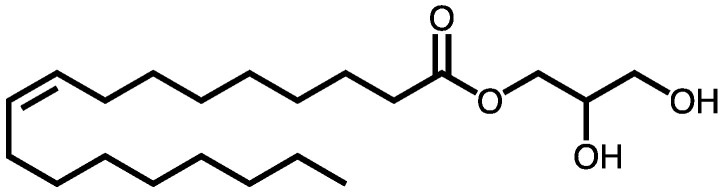
Chemical structure of monoolein (MO).

Monoolein (MO) is a glyceryl 1-monoester of oleic acid (Figure 1), and is one of the most studied skin penetration enhancers [4]. MO penetrates the lipid bilayers of the SC, leading to a disruption of the lamellar structure and an increase in the lipid fluidity [5,6], similar to the skin penetration enhancing effect of oleic acid [7,8]. MO is a non-toxic and biodegradable surface-active compound. Daily use causes no skin irritation when small amounts are applied. However, because of the high lipophilicity of MO, it has been used in combination with polyhydric alcohols such as propylene glycol [9,10,11], or in liquid crystal systems [12,13], and aqueous dispersions of liquid crystals [14,15]. Recently, an oil-based transdermal peptide vaccine delivery system has been reported, using a reverse micelle system composed of water, MO, isopropyl alcohol, and isopropyl myristate (IPM) (2/18/20/60, *w*/*w*) [16]. The reverse micellar system highly efficiently delivered an antitumor peptide vaccine through mouse skin [16], with the assistance of IPM that is also known as an efficient skin penetration enhancer [17,18]. The peptide was delivered to the dendritic cells in the skin at levels greater than three-fold that achieved using a phosphate buffered saline (PBS) delivery vehicle, and successfully suppressed a tumor in the skin. Despite the high drug delivery efficiency, the reverse micelle system is not applicable to protein vaccine delivery due to the denaturation effect of isopropyl alcohol on proteins.

Recently, the bioactive molecules were administered as powders dispersed in the delivery vehicle, to prevent the drug denaturation and to achieve the efficient delivery. The crystal reservoir technology employed in the tulobuterol patch (Hokunalin Tape) enables sustained drug release at a constant level, using a mixture of dissolved tulobuterol and tulobuterol crystals [19]. In other studies, powder proteins and antigens were dispersed in a deep eutectic solvent [20], and loaded in a dissolving microneedle [21], or deposited on an occlusive patch [21]. The Viaskin occlusive patch utilizes sweat and water vapor from the skin, and antigens deposited on patches dissolve into this moisture, and permeate through the SC [22]. The system is expected to be an attractive alternative to oral immunotherapy systems for food allergies such as peanut allergies.

In the current study, we have developed a needle-free protein delivery system composed of protein dispersion in an oil vehicle to facilitate the skin permeation of proteins from powder in an occlusive patch. Factors that could affect the skin permeation efficiencies of proteins were examined, such as the MO concentration, the zeta potential, and the hydrodynamic diameter of proteins. The IPM dispersion of an antigen model protein, ovalbumin from chicken egg white (OVA), was applied to shaved mouse back skin. As a result of the skin permeation enhancing effect of MO, the oil-based patch system elicited a high level of serum antigen-specific antibodies.

## 2. Materials and Methods

### 2.1. Materials

OVA (43 kDa), FITC-conjugated cholera toxin subunit B pentamer (11.6 kDa each), lysozyme from chicken egg white (14 kDa), and FITC-conjugated dextrans (4 k, 20 k, 70 k, 500 kDa) were purchased from Merck (Darmstadt, Germany). Horseradish peroxidase (44 kDa) and bovine serum albumin (66 kDa) were obtained from FUJIFILM Wako Chemicals (Osaka, Japan). Green fluorescent protein (27 kDa) was a generous gift from Prof. K. Minamihata, Kyushu University. N-terminal 5-FAM-labeled peptide (LLDAQSAPLRVYVEELKP, 2 kDa) was custom synthesized at GenScript (Tokyo, Japan). IPM and trypsin (0.5 g/L)-ethylenediaminetetraacetic acid (0.53 mmol/L) solution were purchased from Nacalai tesque (Kyoto, Japan). MGOL-70 was kindly supplied by Nikko Chemicals (Tokyo, Japan). Frozen YMP skin was obtained from Charles River Laboratories (Wilmington, MA, USA) and stored at −80 °C until use.

### 2.2. Animals

Female BALB/c mice (six week old) were purchased from Kyudo (Saga, Japan), and maintained under pathogen-free conditions. All animal experiments were approved by the local Ethics Committee for Animal Experiments (A29-261-0, approved date: 2017-06-16).

### 2.3. Physical Properties of Biomacromolecules

Proteins and FITC-dextrans (0.1 mg/mL, each) were dissolved in PBS. Hydrodynamic diameters and zeta potentials were measured using a Zetasizer NanoZS (Malvern; Malvern, UK). 

### 2.4. Preparation of Liquid-Based Patch Apparatus Carrying OVA Powder

OVA was dissolved in Milli-Q water (10 mg/mL) and a 100 μL aliquot was added in a 1.5-mL tube, and then freeze-dried overnight. Liquid MGOL-70 (40 °C, 25 mg) was added to the freeze-dried OVA powder (1 mg), and they were mixed by vortexing for 10 s. Then, dehydrated IPM (25 °C, 1 mL) was added to the OVA−MGOL-70 dispersion and mixed well using a vortex mixer for 60 s to obtain an OVA dispersion in MO and IPM (OVA/MO/IPM = 1/25/850, *w*/*w*).

The resulting OVA dispersion (50 μL) was penetrated into a polyurethane foam sheet (1 × 1 cm^2^) (Battlewin Underwrap, Nichiban; Tokyo, Japan), supported by a waterproof-breathable backing with an acrylic adhesive (2.5 × 2.5 cm^2^) (FC Waterproof Film, Hakujuji; Tokyo, Japan) to prepare a patch. 

For the fluorescence imaging experiment of OVA in the skin, OVA was labeled with Cy5 using a Cy5 mono-reactive dye pack kit (GE Healthcare; Buckinghamshire, England) according to the manufacturer’s instructions and was loaded in the patch in the same manner. 

### 2.5. Ex-Vivo Skin Permeation Tests

The ex-vivo skin permeability of the OVA was examined using Franz diffusion cells (receptor volume; 5 mL, effective diffusion area; 0.785 cm^2^). The YMP skin stored at −80 °C was thawed and the subcutaneous fats and tissues were carefully removed using a scalpel. Then, the skin was cut into 1.5 × 1.5 cm^2^ pieces and placed onto the receptor compartment. The receptor compartment was filled with PBS and maintained at 32 °C. FITC-labeling of proteins was performed as previously reported [23]. FITC-OVA dispersion liquid (1 mg/mL, 200 μL) was loaded into the donor compartment. After 24 h, the receptor medium was collected, and each piece of skin was rinsed with Milli-Q water (5 mL), 80% ethanol (5 mL), then Milli-Q (5 mL) again, and then wiped with a paper. The skin was cut into eight pieces, and the FITC-OVA was extracted in PBS (1 mL) through vortexing (1800 rpm) for 16 h at room temperature. FITC-OVA concentrations in the receptor media and the skin extracts were determined using a fluorescence microplate reader from BioTek Instruments with excitation and emission filters of 480/20 and 520/20 (Winooski, VT, USA). Concentrations of FITC-labeled proteins and FITC-dextrans in the skin extracts were determined in the same manner.

### 2.6. OVA Stability in Oil Dispersion

The OVA dispersions (1 mg/mL) with and without MGOL-70 (25 mg/mL) were prepared and stored for 24 h at room temperature. An aliquot (200 μL) was carefully added to PBS (1 mL) and stirred with a magnetic stirrer for 24 h to let the OVA molecule dissolve. The protein concentration in PBS was determined at 280 nm and adjusted to be 0.1 mg/mL with PBS. The secondary structures of OVA in the PBS (0.1 mg/mL) were analyzed using a circular dichroism spectrometer J-725G (JASCO; Tokyo, Japan) at 25 °C with a 1 mm cell. The percentage of the α-helical structure was calculated using the equation reported by Cheng and Yang [24].

### 2.7. Analyses of SC Structure

The SC was isolated from YMP skin as reported previously [25,26]. Briefly, the commercially obtained full-thickness YMP skin was sectioned to a thickness of 2–3 mm using a scalpel and cut into 1 × 1 cm^2^ pieces. The skin pieces were placed in a plastic dish and immersed in a trypsin (0.5 g/L)-EDTA (0.53 mmol/L) solution. The skin was incubated overnight at 30 °C, and then the SC was peeled off. The SC was rinsed with Milli-Q water, air-dried, and placed on a glass plate, SC side up. A patch was applied to the SC surface for 24 h at 32 °C. After removal of the patch, the SC sample was rinsed with Milli-Q water, 80% ethanol, and then Milli-Q water (5 mL each) again, and then immediately wrapped in a plastic film and stored on ice until analysis. 

Small-angle X-ray scattering (SAXS) patterns were recorded using a NANOSTAR system (Bruker; Billerica, MA, USA), with CuKα radiation at 50 kV and 100 mA. The SC sample was attached to the film holder with Kapton tapes. The film holder was equipped with a water heating system adjusted to 32 °C. SAXS patterns were scanned for 2 h under atmospheric pressure. 

Fourier transform infrared (FT-IR) spectra were measured using a Spectrum Two equipped with a Universal Attenuated Total Reflectance accessory (Perkin Elmer, Waltham, MA, USA). The FT IR spectra of SC were measured at a resolution of 2 cm^−1^, with 64 scans at room temperature. An air background spectrum was withdrawn from each spectrum. 

### 2.8. In Vivo Skin Permeation Tests

Mouse hair was removed using depilatory cream 24 h prior to OVA administration. The patch apparatus was applied onto the back skin (n = 3). After 24 h, the patches were removed and wiped with isopropanol, and OVA diffusion was observed using an IVIS Lumina II (Summit Pharmaceuticals, Tokyo, Japan). Mice were sacrificed and the skin areas where the samples were attached were collected. The skin was cut into halves and confocal images were obtained using a Confocal Laser Scanning Microscope LSM700 with a 20× objective lens (Carl Zeiss, Oberkochen, Germany). Half of the skin was embedded in Tissue-tek O.C.T. compound, frozen in liquid nitrogen, and then sectioned at a 16-μm thickness using a cryostat CM1860UV (Leica, Wetzlar, Germany). The skin sections were observed using a fluorescence microscope BZ-9000 with a 20× objective lens (Keyence, Osaka, Japan).

### 2.9. Antibody Responses in Mice

Freeze-dried OVA (1 mg) was dispersed in MGOL-70 (25 mg) and then IPM (0.5 mL) for the immunization of the mice. An aliquot (2 mg/mL, 50 μL) was loaded onto the patch apparatus, and then placed onto the mouse back skin for 24 h, once a week for 3 w (n = 6). A total of 2 w after the final administration, blood samples were collected for sera. PBS solutions of OVA (2 mg/mL, 50 μL) were similarly applied onto the mouse back skin as controls. A serum OVA-specific IgG titer was measured as previously reported [27].

Statistical analysis: OVA-specific IgG levels were analyzed for the distribution of the oil-based delivery system using Student’s t test (* *p* < 0.05).

## 3. Results

### 3.1. Ex Vivo Permeation Test Using Yucatan Micropig (YMP) Skin

#### 3.1.1. Effect of MO concentration

**Figure 2:**
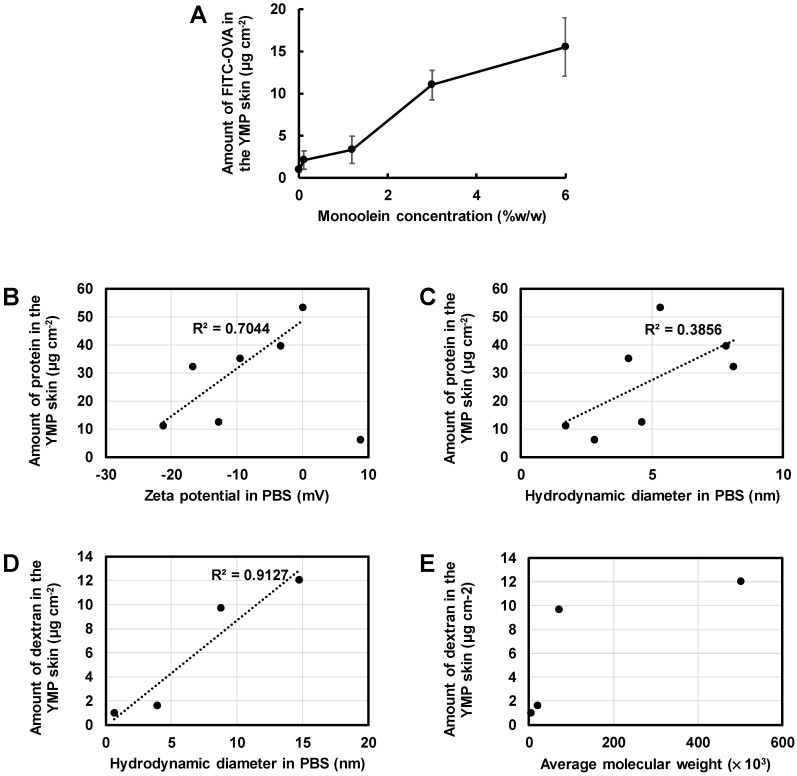
Efficiencies of skin permeation of proteins and dextrans (*n* = 3). (**A**) The skin permeability of fluorescein isothiocyanate (FITC)-labeled ovalbumin (OVA) with respect to the concentration of monoolein (MO) dissolved in isopropyl myristate (IPM). (**B**,**C**) The skin permeabilities of proteins with respect to zeta potential (**B**), and hydrodynamic diameter (**C**) at an MO concentration of 3% *w*/*w*. (**D**,**E**) The skin permeabilities of FITC-labeled dextrans in relation to hydrodynamic diameter (**D**), and average molecular weight (**E**) at an MO concentration of 3% *w*/*w*. All values are shown in the supporting information.

MO is a well-known amphiphile that facilitates the skin permeation of small molecule pharmaceuticals. We examined the ability of MO (MGOL-70) to function as a skin penetration enhancer of hydrophilic biomacromolecules, proteins and dextrans. The skin permeability of OVA was increased depending on the concentration of MO up to 6% *w*/*w* (Figure 2A). Less OVA (1 μg/cm) permeated the skin when the PBS solution (1 mg/mL) was applied. OVA was not detected in the receptor media even after 24 h of incubation. 

#### 3.1.2. Factors Influencing Passive Delivery of Macromolecules

The skin permeabilities of various proteins were examined at an MO concentration of 3% *w*/*w*. The skin permeation efficiency was strongly affected by the zeta potential of the protein, rather than by the hydrodynamic diameter of the protein (Figure 2B,C). The amounts of protein undergoing skin permeation from oil-dispersions and the PBS solutions are shown in Figure S1. The proteins were not detected in the receptor media in any groups. The results drove us to examine the skin permeability of a series of fluorescein isothiocyanate (FITC)-dextrans at varying average molecular weights and hydrodynamic diameters. All FITC-dextrans examined here displayed a zeta potential of −5.0 in PBS. The skin permeation efficiency of FITC-dextrans increased along with their hydrodynamic diameter in PBS, rather than with the average molecular weight (Figure 2D,E).

### 3.2. OVA Stability in Oil Vehicle

**Figure 3:**
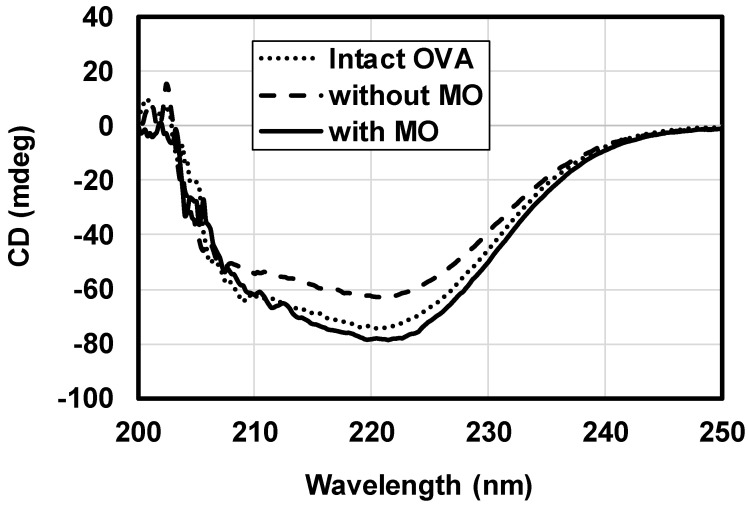
Circular dichroism spectra of intact ovalbumin (OVA) in PBS, and the OVA released from the oil dispersions with or without monoolein (MO) (*n* = 3).

According to a previous report, the secondary structure of crystal OVA (PDB code: 1OVA) is composed of 30.8% α-Helix, 31.3% β-Strand, and 16.4% Turn [28]. In the present report, we examined the effects of IPM and MO in the OVA dispersions. OVA was released into PBS from the IPM dispersions with or without MO. According to the absorbance at 280 nm, 99% (IPM with MGOL-70) and 98% (IPM) of the OVA was released from each dispersion. Circular dichroism spectra of the released OVA were assessed and compared with those of the intact OVA (Figure 3). The negative minimum at 221 nm derived from the α-helical structure slightly increased in the spectrum for OVA released from IPM without MO. The percentages of an α-helical structure calculated from the spectra were 27%, 28%, and 23%, in intact OVA, and in OVA released from IPM with MO, and from IPM without MO, respectively. The spectral change in OVA was small for the IPM dispersion with MO, suggesting that the helical structure was preserved for at least 24 h.

### 3.3. Structural Change in the Stratum Corneum

**Figure 4:**
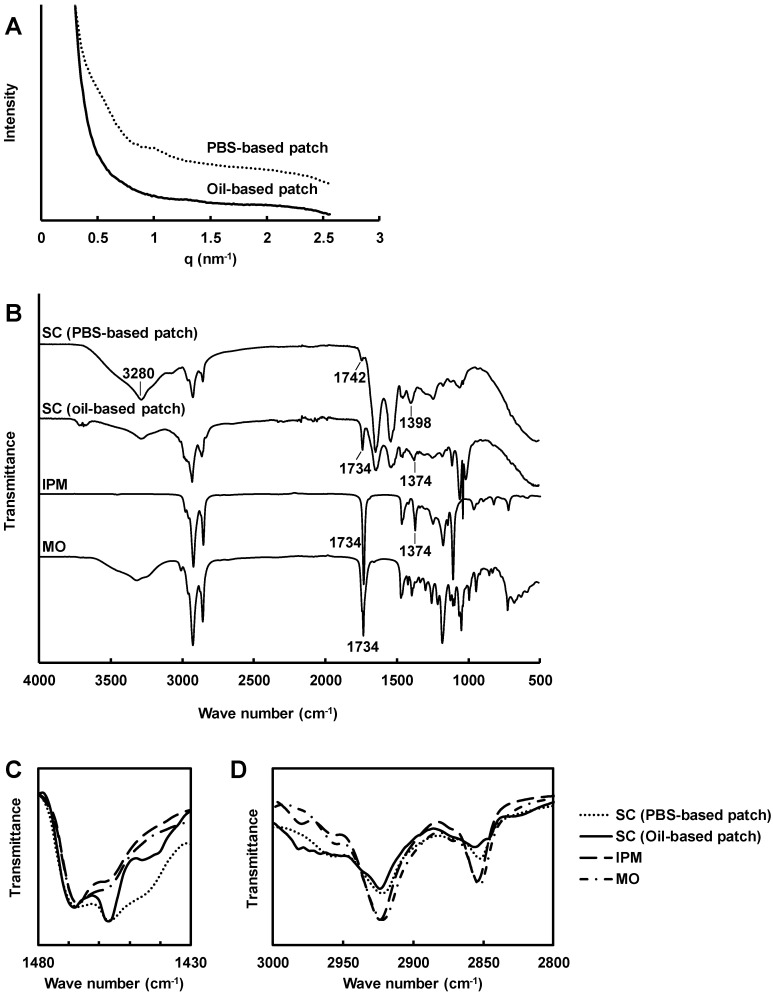
Structural analysis of stratum corneum (SC) treated with a PBS-based ovalbumin (OVA) patch, and an oil-based OVA patch for 24 h at 32 °C (*n* = 4). (**A**) Small-angle X-ray scattering patterns of SC samples. (**B**–**D**) Attenuated total reflection infrared spectra of SC samples, isopropyl myristate (IPM), and monoolein (MO) from 500 cm^−1^ to 4000 cm^−1^ (**B**), the CH_2_ bending region derived from ceramides (**C**), and the CH_2_ symmetric and asymmetric stretching regions (**D**).

The SC was isolated from YMP skin and applied to the patch for 24 h. The small-angle X-ray scattering pattern of the SC treated with PBS-based patches showed typical lamellar patterns of a 5.5-nm period (Figure 4A). The peaks diminished after application of the oil-based patches, which indicated the dehydration and oil penetration in the SC. The ATR-IR spectra showed a decline in a broad peak at 3280 cm^−1^ derived from water molecules through treatment with the oil-based patches (Figure 4B). As a result of dehydration, narrow peaks from 3660 cm^−1^ to 3710 cm^−1^ were observed, which are the stretching vibrations of non-bonded O–H groups. The peak frequencies of 1742 cm^−1^ (νC=O) and 1398 cm^−1^ (δCH_2_) disappeared [29], as a result of the extraction of lipids in the SC. The fingerprint patterns from 1500 cm^−1^ to 800 cm^−1^ in the MO and IPM spectra were observed in the spectrum of the SC treated with the oil-based patches, indicating penetration of these substances into the SC. The peaks from 1440 cm^−1^ to 1470 cm^−1^ became sharp, indicating the disordering of ceramides from orthorhombic perpendicular packing (Figure 4C) [30]. The CH_2_ symmetric and asymmetric stretching peaks at around 2850 cm^−1^ and 2925 cm^−1^, respectively, are significant indicators of the lipid fluidity in the SC. A little change in the peak frequency derived from symmetric νCH_2_ was observed between the SC treated with the PBS-based patch and with the oil-based patch. No frequency change was observed in the peak derived from asymmetric νCH_2_. However, sharpening of the peak indicated penetration of IPM into SC after treatment with the oil-based patch (Figure 4D).

### 3.4. In Vivo Skin Permeation Test on Mice

**Figure 5:**
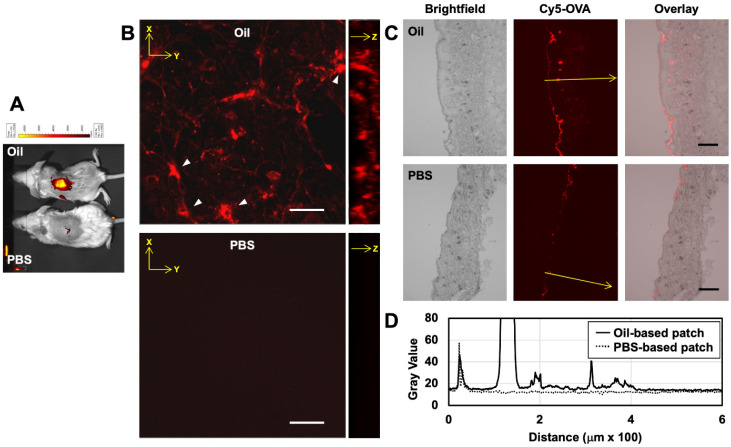
Transdermal delivery of Cy5-labeled ovalbumin (OVA) on mouse back skin using an oil-based patch (Oil) and a PBS-based patch (PBS) (*n* = 3). (**A**) Diffusion of the Cy5-OVA on the mouse back. (**B**) Confocal laser scanning fluorescence microscope images of mouse back skins treated with an oil-based patch and a PBS-based patch. OVA-captured cells are indicated by white triangles. (**C**) Fluorescence microscope images of mouse back skins sectioned at a 16-μm thickness. (**D**) Pixel intensity profiles of the positions are shown by arrows. Bars indicate 50 μm (**B**) and 200 μm (**C**).

Patches carrying Cy5-OVA were applied to mouse back skin for 24 h, and the OVA diffusion was assessed using IVIS in-vivo imaging equipment. Most of the OVA permeated vertically at the area where the patch was applied, and a slight horizontal diffusion was observed (Figure 5A). No signals were detected from the lymph nodes, the spleen nor any other organs. Then, the excised skin was observed using a confocal fluorescence laser scanning microscope (Figure 5B). Cy5-OVA was detected inside several cells as well as the intercellular spaces. Such antigen uptake by antigen presenting cells (APCs) was not observed when the PBS solution of OVA was applied to the mouse skin in the same manner. The excised skin was further sectioned and observed using a fluorescence microscope (Figure 5C). In the skin sections, OVA mostly penetrated through the SC because of the action of MO in collapsing the SC lipid bilayer. Penetration of OVA was also observed in hair follicles and through to the upper dermis (Figure 5D).

### 3.5. Immune Response in Mice

**Figure 6:**
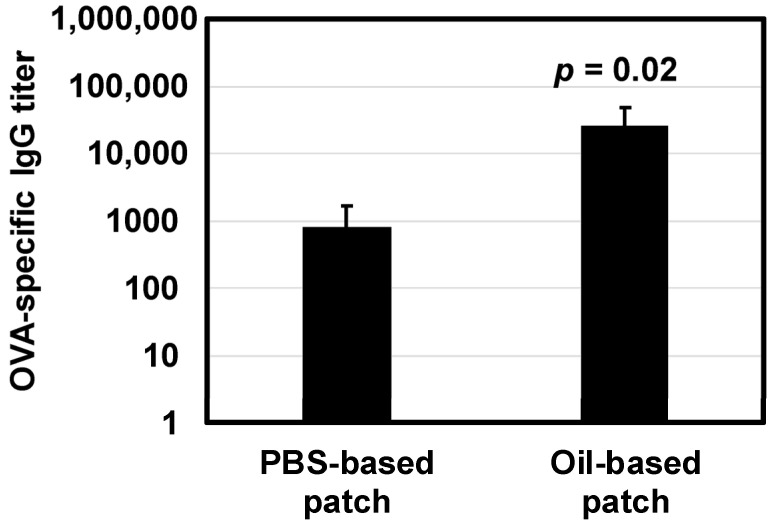
Antigen-specific antibody responses in mouse sera treated with a PBS-based ovalbumin (OVA) patch, and oil-based OVA patches (*n* = 6). Data was showed as an average and a standard deviation.

Mice were immunized using OVA-carrying patches three times. Blood samples were collected 2 weeks after the final booster. This time schedule was determined because OVA-specific IgG levels were highest at this time point in a previous transdermal administration of OVA using a solid-in-oil nanodispersion system [27]. The OVA-specific IgG titer was significantly higher in the group that received the oil-based patch than the one that received the PBS-based patch (Figure 6).

## 4. Discussion

MO, also known as glyceryl monooleate, is an amphiphilic polar lipid, and has been widely used as an emulsifier, moisturizer, and thickening agent in foods, cosmetics, and pharmaceuticals [31]. It is non-toxic and biodegradable [4]. For topical daily use, MO causes minimal reversible skin irritation at up to 5% *w*/*w* of the total ingredients in cosmetic products [32]. MO has a great potential in transdermal drug delivery systems because it can increase topical and transdermal drug penetration. However, to the best of our knowledge, previous investigations have focused on the topical and transdermal delivery of small molecule pharmaceuticals and peptides (~2 kDa), such as 5-aminolevulinic acid [33], doxorubicin [10], and cyclosporine A [9]. In a previous report, MO increased the permeation of cyclosporin A, a cyclic peptide, into porcine ear skin up to 70% using a water-propylene glycol system [9]. Furthermore, the amount of cyclosporin A in the receptor phase increased up to an MO concentration of 10% *w*/*w*, while it decreased at MO concentrations above 10% *w*/*w*. Another group reported that the steroid hormone progesterone was delivered into the skin by MO, and the permeability of progesterone decreased when the concentration of MO was higher than 5% *w*/*w* [11]. In our patch system, MO enhanced the skin permeation of OVA linearly up to 6% *w*/*w*.

MO is thought to increase the fluidity of SC membrane lipids and allow drugs to permeate through the defects in the lipid bilayer [5,6]. Given that MO facilitated the skin permeation of large hydrophilic molecules in our oil-based patch system, we examined the factors that influenced the passive permeation of proteins. Conventionally, successful transdermal delivery of small molecules has been achieved with low molecular masses (<500 Da) and moderate lipophilicity (Log P = 1–3) through passive absorption [34,35]. In our oil-based system, protein permeability was more affected by the zeta potential of the proteins rather than their hydrodynamic diameter. Since the skin surface is negatively charged, proteins with a lower zeta potential may not be able to diffuse efficiently through the skin [36]. Proteins with a positive zeta potential also did not permeate through the skin. At an MO concentration of 3% *w*/*w* and at 24 h of incubation, the proteins were not detected in the receptor medium; however, this is preferable in some cases such as antigen immunotherapy. In epicutaneous antigen immunotherapy, antigens are delivered only to the epidermis and are inhibited from entry to the dermis to prevent antigen delivery to the systemic circulation and thereby avoid acute severe immune reactions [21,22].

Dextran is a polysaccharide that, unlike proteins, has no specific secondary nor tertiary structures. The zeta potential of FITC-dextran in PBS was approximately −5.0 regardless of the average molecular weight. In the case of FITC-dextran, macromolecules with a higher particle size could permeate efficiently into the skin. According to the report from Potts and Guy, the permeability coefficient is proportional to the diffusion coefficient and inversely proportional to the diffusion pathlength [37]. Monoolein was supposed to have created cavities and channels of various lengths and diameters. As a result, dextrans with smaller hydrodynamic diameters would have been trapped in the small cavities and could not penetrate the SC efficiently.

To deliver large molecules such as proteins, pharmaceutical stability is of concern, as well as other factors such as half-life, bioavailability, and toxicity. A circular dichroism (CD) spectrum is an indicator of the secondary structure of a protein [38]. In the present study, OVA was released from the oil vehicle into PBS and the spectra were then measured. In the absence of MO, the helical content in the OVA was reduced. When MO was present, both sheet and helical content were increased, although the spectral change was minor. The above results show that MO can interact with the OVA molecule; however, the secondary structures were retained in the oil vehicle. As with protein released from a deep eutectic solvent [20], it was possible that the OVA secondary structure was reconstituted after it was eluted into the PBS.

Although MO did not denature the OVA, it was possible that it affected the lipids in the SC. Based on the small angle X-ray scattering patterns, MO affected the lamellar structure in the SC at 3% *w*/*w*. In previous studies, the periodic X-ray diffraction pattern derived from the SC disappeared following dehydration [39], and through pooling of lipophilic penetration enhancers such as d-limonen in the SC [25]. In the current study, dehydration and the uptake of IPM and MO were observed in the FT–IR spectrum of SC treated with the oil-based patch. In addition, extraction of free fatty acids was suggested by the disappearance of two peaks in the FT–IR spectra [26,40]. The peak frequencies from 1440 cm^−1^ to 1470 cm^−1^ derived from the symmetric CH_2_ bending indicated disordering of the ceramide orthorhombic perpendicular packing following oil-based patch application [30]. The symmetric and asymmetric CH_2_ stretching region from lipids (2850–2925 cm^−1^) indicates the alignment and the fluidity of the lipids [41]. The shift of the peak derived from the symmetric CH_2_ stretching occurred from 2852 cm^−1^ to 2856 cm^−1^ indicated an increase of the lipid fluidity in the SC caused by monoolein. No shift was observed in the asymmetric CH_2_ stretching peak, however, sharpening of the peak at 2928 cm^−1^ indicated infiltration of IPM into the SC.

As mentioned above, MO is non-toxic. However, repeated overuse may cause skin irritation, as with oleic acid [4]. In the current study, the oil-based occlusive patch system was applied to mouse back skin for 24 h. After removal of the patch, no erythema was observed on the skin. IVIS imaging revealed that the OVA in the oil-based patch system had vertically permeated into the mouse back skin. Compared with the PBS-based patch, the oil-based patch could deliver more OVA into the skin. Confocal images showed that OVA had reached the interior of some cells, which had corresponding sizes and shapes to the APCs shown in a previous report [21]. In the images of skin sections, OVA was mainly found in the SC, and a smaller amount was found around hair follicles and in the upper dermis. Unfortunately, APCs were not confirmed in the immunostained sections (data not shown). In a previous study using mice that secrete MHC II fused with enhanced green fluorescent protein, APCs assembled around OVA delivered using microneedles after 24 h [21]. Thereafter, APCs eventually migrated into the subcutaneous tissues over several days. Since the SC seemed to act like a reservoir of OVA in our system, APCs could have captured the antigens and migrated into the deep skin following a similar time course as that in the previous research.

The oil-based patch system elicited significantly higher levels of antigen-specific IgG antibodies than the aqueous-based patch system. The skin thickness differs between mice and humans, and the human skin thickness and structure are more similar to those of the YMP skin. Since OVA permeated through to the middle of the dermis in the YMP skin, and not to the receptor phase after 24 h, the efficiency of the oil-based patch on human skin needs to be investigated. However, APCs exist in the human epidermis, and some techniques, such as epicutaneous immunotherapy, successfully achieved modification of antibody responses by delivering the antigen proteins to the epidermis [21,22]. A transdermal vaccine delivery system using a needle-free patch carrying MO in an IPM solution is a potent approach for an easy-handling and convenient immunization.

## 5. Conclusions

An MO-assisted oil-based occlusive patch to deliver antigen powder to the skin was investigated. The protein permeability increased along with increasing MO concentrations in the IPM vehicle, indicating the skin permeation enhancement of MO. The proteins with higher negative zeta potentials more easily permeated into the skin. The effect of MO in our oil system was suggested to be limited to SC. Any serious adverse events were not observed during patch application on mouse back skin for 24 h. The oil-based system induced significantly higher levels of antigen-specific IgG than that of the aqueous system, indicating the potential of the oil-based patch system for a non-invasive transdermal vaccine delivery. 

## Supplementary Materials

The following are available online at https://www.mdpi.com/1999-4923/12/9/814/s1, Figure S1: Physical properties of proteins examined in the skin permeability test and the amounts that permeated Yucatan micropig skin using oil-dispersion systems and PBS solutions (1 mg/mL, each).
