# Supplementary material for: Monoolein Assisted Oil-Based Transdermal Delivery of Powder Vaccine
Source: Pharmaceutics. 2020 Aug 27;12(9):814. doi: 10.3390/pharmaceutics12090814 (PMC7558954; doi:10.3390/pharmaceutics12090814)
Supplement: Supplementary file 1 [file pharmaceutics-12-00814-s001.pdf]

pharmaceutics

Supplementary Material

Monoolein Assisted Oil-Based Transdermal Delivery of Powder Vaccine

Momoko Kitaoka, Atsushi Oka and Masahiro Goto

Protein	Molecular weight (kDa)	Size in PBS (nm)	Isoelectric point	Zeta potential (mV)	Permeability		Determination method
					PBSsol (µg cm^{-2})	oil dispersion (µg cm^{-2})	
**Ovalbumin**	**43**	**4.6**	**4.9**	**-12.8**	**0.41**	**12.5**	**FITC**
**Horseraddish peroxidase**	**44**	**5.3**	**6**	**0.007**	**0.98**	**53.3**	**3,3',5,5'-tetramethylbenzidin**
**Bovine serum albumin**	**66.5**	**8.1**	**4.7**	**-16.7**	**3.6**	**32.1**	**FITC**
**Enhanced green fluorescent protein**	**29**	**4.1**	**6.1**	**-9.52**	**4.2**	**35**	**Fluorescence from protein**
**Cholera toxin subunit B**	**58**	**7.8**	**6.6**	**-3.29**	**9.8**	**39.4**	**FITC**
**N-terminal FAM-Peptide**	**2.041**	**1.7**	**4.7**	**-21.2**	**1.3**	**11.1**	**FAM**
**Lysozyme**	**14.3**	**2.8**	**11**	**8.84**	**4**	**6**	**FITC**

Figure S1. Physical properties of proteins examined in the skin permeability test and the amounts that permeated Yucatan micropig skin using oil-dispersion systems and PBS solutions (1 mg/mL, each).

© 2020 by the authors. Licensee MDPI, Basel, Switzerland. This article is an open access article distributed under the terms and conditions of the Creative Commons Attribution (CC BY) license (http://creativecommons.org/licenses/by/4.0/).
